# Study designs of randomized controlled trials not based on Chinese medicine theory are improper

**DOI:** 10.1186/1749-8546-4-3

**Published:** 2009-02-25

**Authors:** Jian Yan, Veronica F Engle, Yuxin He, Yan Jiao, Weikuan Gu

**Affiliations:** 1 Molecular Resource Center, University of Tennessee Health Science Center, Memphis, TN 38163, USA; 2 Department of Primary Care Nursing and Department of Preventive Medicine, University of Tennessee Health Science Center, Memphis, TN 38163, USA; 3 Acupuncture & Chinese Medical Center, Austin, TX 78757, USA; 4 Department of Orthopedics Surgery, University of Tennessee Health Science Center, Memphis, TN 38163, USA

## Abstract

Current biomedical research methods to evaluate the efficacy of Chinese medicine interventions are often conceptually incompatible with the theory and clinical practice of Chinese medicine. In this commentary, we (1) highlight the theory and principles underlying Chinese medicine clinical practice; (2) use ginseng as an example to describe clinical indications in Chinese medicine; (3) propose a framework guided by Chinese medicine theory for the evaluation of study designs in Chinese medicine research; and (4) evaluate 19 randomized, double-blind, placebo-controlled trials of ginseng. Our analysis indicates that all 19 trials with both positive and negative results confirm the specific effects of ginseng indicated by Chinese medicine theory. Study designs guided by Chinese medicine theory are necessary to validate and improve future randomized controlled clinical trials in Chinese medicine.

## Background

Chinese medicine remains popular in China where traditional herbal preparations are estimated to account for 30–50% of the total medicinal consumption [1]. Chinese medicine has also been gaining popularity in the West [1-3]. However, Chinese medicine lacks funding and leading scientists to conduct scientific research (e.g. randomized controlled trials) [4].

The study of ginseng provides an example of the research challenges in Chinese medicine. Highly valued in the Chinese medicine classics and widely used in China for more than two thousand years, ginseng has yet to prove its safety and efficacy through clinical trials [5,6], which, many investigators believe, may be attributed to a paradigm conflict and the poor quality of some clinical trials [7,8].

We found that this paradigm conflict may be resolved by using study designs guided by Chinese medicine theory.

### Chinese medicine theory

Chinese medicine is a syndrome-oriented holistic medical system that is conceptually distinct from its Western counterpart. According to Chinese medicine theory, a syndrome is a group of associated signs and symptoms described in terms of Yin and Yang, *Qi*, and *Xue *(blood) [9]. All illnesses fall into eight principal categories used to guide the prevention and treatment of illnesses [10]. These categories are Yin and Yang, *Biao *(exterior) and *Li *(interior), *Han *(coldness) and *Re *(heat), and *Xu *(deficiency) and *Shi *(excess). Western medicine, however, views a disease or syndrome as pathological changes of specific biological processes [1]. As a result, the syndromes in Chinese medicine do not always correspond with Western classifications of diseases and syndromes. For instance, hypertension may be related to syndromes of *Gan *(liver) Yang ascending, Yin deficiency of liver and kidney, flaming liver fire, stagnation of phlegm, *Xue *stasis and/or dual Yin/Yang deficiency [11]. Conversely, *Qi*-deficiency syndrome is related to chronic obstructive pulmonary disease [12], lung cancer [13], coronary heart disease [14] and persistent allergic rhinitis [15].

### Herbal medications

In Chinese medicine, medicinal herbs are categorized according to the concepts of Yin, Yang, *Qi*, *Xue*, *Jing *(essence) and *Jin *(body fluid) [16]. In general, 'tonics' are used to treat deficiency and 'clear-ups' are used to treat excess [9].

Considered the premium *Qi*-tonifying herb to treat various illnesses [16], ginseng is thought to have the major indications as follows:

(1) Impalpable pulse caused by severe *Qi*-deficiency;

(2) Shortness of breath, feeble voice, spontaneous sweating and a weak pulse caused by *Fei *(lung) *Qi*-deficiency;

(3) Fatigue, anorexia and loose bowels caused by *Pi *(spleen) *Qi*-deficiency;

(4) Fever and strong thirst caused by *Qi*-deficiency;

(5) Palpitation, insomnia and forgetfulness caused by dual deficiency of *Qi *and *Xue*.

### Study design compatible with Chinese medicine theory

#### Research topics

Instead of evaluating the efficacy of ginseng in all patients suffering from a single disease, researchers should focus on those patients with *Qi*-deficiency syndrome. *Qi*-deficiency causes decreased visceral functions and lowered immune resistance, leading to various diseases. The manifestations of *Qi*-deficiency include lassitude, shortness of breath, feeble voice, dizziness, spontaneous perspiration, susceptibility to cold, pale tongue and weak pulse [10].

#### Participants

Chinese medicine practitioners prescribe herbal medications to rectify disharmony in a patient's system [16]. Healthy individuals should not participate in treatment groups in Chinese medicine studies. This explains the negative results from the ginseng studies in which healthy individuals participated [17-21].

Ginseng is a *Qi*-tonifying herb to treat five major syndromes [16] caused by *Qi*-deficiency. Therefore, we argue that only studies in which participants are diagnosed with *Qi*-deficiency are valid to evaluate ginseng's efficacy [22-25].

#### Herbal species

While at least eight species of ginseng are commercially available [26], only two major species, namely *Panax ginseng *(Chinese or Korean ginseng) and *Panax quinquefolius *(American ginseng), are used as medicinal herbs worldwide. According to Chinese medicine theory, the properties and functions of these two species are quite different [16]. While *P. ginseng *enhances Yang, *P. quinquefolius *nourishes Yin. A search for randomized controlled trials of ginseng in PubMed (7 September 2008) found that about one-third of the studies did not mention the ginseng species used and that very few studies addressed the species issue.

#### Herbal quality

Herbal quality may affect research results. Different batches of *P. ginseng *[27,28] or *P. quinquefolius *[29] produced opposing study results respectively on acute postprandial glycemia. The primary active ingredients in ginseng are ginsenosides. G115, a ginsenoside-based standardized extract of *P. ginseng*, may help assess the efficacy and safety of ginseng. In fact, G115 was used in most *P. ginseng *(single herb) trials reviewed in this paper.

#### Herbal formulae

In Chinese medicine, herbs are often formulated to achieve increased therapeutic effects and reduced toxicity or side effects [16]. Results from clinical trials on herbal formulae confirm this practice. A Japanese trial found that a 7-herb formula was effective in preventing liver cancer in cirrhosis patients [30]. Two British trials showed that a 10-herb formula was effective in treating a severe atopic eczema [31,32]. No single herbal ingredient explains the efficacy in these studies [33]. Furthermore, ginseng herbal formulae were shown to be effective in treating chronic pulmonary disease [22,34], congenital heart disease [35,36], mild cognitive impairment [37], coronary heart disease [38] and nasopharyngeal carcinoma [39].

#### Herbal safety

Certain Chinese medicine herbs are toxic and others may have adverse effects when used improperly [16]. A condition known as the ginseng abuse syndrome is characterized by heart palpitations, heaviness in the chest, high blood pressure, dizziness, insomnia, agitation, restlessness, nausea, vomiting, abdominal pain and/or bloating, diarrhea, possible upper digestive tract bleeding, edema, and red skin rash [40]. Most of these reported adverse effects are common manifestations of *Qi*-excess and *Qi*-stasis. While all clinical trials should document adverse effects, only one trial did do so [41].

### Re-examination of equivocal ginseng trial results

To exemplify our framework of experimental study design, we searched and analyzed randomized controlled trials of ginseng in PubMed. The inclusion criteria were single herb ginseng trials with a sample size of ≥20. We selected trials of single herb ginseng because the majority of the trials belonged to this category. Nineteen clinical trials were selected for analysis according to the inclusion criteria (Table 1) [42-46]. Most of the trials were considered good based on a trial quality evaluation scale [47].

**Table 1:** Summary of results from single herb ginseng clinical trials

	Trial quality*	Research topic	Participants (*n*)	Herb species	Chinese medicine theory	Reference
**Negative results**						
Allen JD *et al*. (1998)	4	Exercise performance	Healthy young (28)	*P. ginseng*	No *Qi*-deficiency	[17]
Cardinal BJ *et al*. (2001)	4	Psychological well-being	Healthy young adults (83)	*P. ginseng*	No *Qi*-deficiency	[18]
Caron MF *et al*. (2002)	3	Cardiovascular function	Healthy adults (30)	*P. ginseng*	No *Qi*-deficiency	[42]
Dowling EA *et al*. (1996)	3	Exercise performance	Highly trained distance runners (20)	*Acanthopanax senticosus*	No Qi-deficiency	[43]
Engels HJ *et al*. (1997)	3	Physiologic and psychological responses	Healthy adults (36)	*P. ginseng*	No *Qi*-deficiency	[21]
Engels HJ *et al*. (2001)	3	Exercise & short-term recovery	Healthy active women (24)	*P. ginseng*	No *Qi*-deficiency	[20]
Engels HJ *et al*. (2003)	3	Physical performance heart rate recovery	Active healthy adults (38)	*P. ginseng*	No *Qi*-deficiency	[19]
Stavro PM *et al*. (2006)	3	Blood pressure and renal function	Hypertension (52)	*P. quinquefolius*	Inappropriate herb species	[49]
Wiklund IK *et al*. (1999)	N/A	Quality of life & physiological parameters	Symptomatic postmenopausal women (384)	*P. ginseng*	Inappropriate herb species	[51]
**Positive results**						
Cicero AF *et al*. (2004)	2	Quality of life	Elderly hypertensive and digitalized (20)	*Acanthopanax senticosus*	Appropriate herb species	[53]
de Andrade E *et al*. (2007)	2	Sexual function	Erectile dysfunction (60)	*P. ginseng*	*Qi*-Deficiency	[44]
Ellis JM *et al*. (2002)	5	Quality of life	Healthy young (30)	*P. ginseng*	Marginal *Qi*-deficiency	[48]
Gross D *et al*. (2002)	N/A	Respiratory function	Chronic Obstructive Pulmonary Disease (COPD) (92)	*P. ginseng*	*Qi*-Deficiency	[22]
Hong B *et al*. (2002)	3	Sexual function	Erectile dysfunction (45)	*P. ginseng*	*Qi*-Deficiency	[45]
Kim JH *et al*. (2006)	3	Quality of life	Cancer (53)	*P. ginseng*	*Qi*-Deficiency	[23]
Liang MT *et al*. (2005)	3	Endurance exercise	Untrained adults (29)	*P. notoginseng*	Appropriate herb species	[54]
McElhaney JE *et al*. (2004)	3	Acute respiratory illness	Sub healthy seniors(198)	*P. quinquefolius*	*Qi*-Deficiency	[24]
McElhaney JE *et al*. (2006)	5	Acute respiratory illness	Sub healthy adults and seniors (43)	*P. quinquefolius*	*Qi*-Deficiency	[46]
Predy GN *et al*. (2005)	5	Cold	Sub healthy adults (323)	*P. quinquefolius*	*Qi*-Deficiency	[25]

*Trial quality evaluation scale [47]

0–2: poor quality

3–5: high quality

N/A: full text unavailable for quality evaluation

#### Research topic

Out of the 19 trials, nine had negative results, ten had positive results, and none targeted ginseng's efficacy on *Qi*-deficiency syndromes.

#### Participants

Both healthy and unhealthy participants were evaluated for the effects of ginseng. Seven out of the nine trials with negative results involved healthy participants, whereas eight out of the ten studies with positive results had participants with *Qi*-deficiency manifested by cancer, impotence and pulmonary disease. Ellis *et al*. [48] investigated the time-dependent effects of *P. ginseng *on the quality of life in a healthy young adult population. In this case, the participants had marginal *Qi*-deficiency as young adults are at the stage of 'gradual filling of *Qi *and *Xue*' [9] according to Chinese medicine theory.

#### Herbal species/safety

The species of ginseng may be a confounding factor in the interpretation of trial results, which is illustrated by four trials as follows (Table 1).

Stavro *et al*. [40] enrolled 52 hypertensive participants to evaluate the long-term effects of *P. quinquefolius *on blood pressure [49]. Long-term use of ginseng was reported to be associated with the development of hypertension, which was refuted by Stavro *et al*. In Chinese medicine practice, however, *P. quinquefolius*, unlike its cousin *P. ginseng*, is in fact used to treat hypertension in some cases.

Wiklund *et al*. [50] reported a trial in which 384 symptomatic postmenopausal women were assessed for the effects of *P. ginseng *on the quality of life and physiological parameters. Postmenopausal symptoms such as hot flashes are often regarded as *Shen *(kidney) Yin-deficiency [51] and are treated with *P. quinquefolius *rather than *P. ginseng*. Moreover, the use of *P. ginseng *in this study was contraindicated and might have produced adverse effects.

Cicero *et al*. [52] studied 20 elderly hypertensive and digitalized patients treated with *Acanthopanax senticosus *(Siberian ginseng) which is a mild *Qi*-tonic for an unspecific feeling of fatigue, a sign of *Qi*-deficiency [53]. Hypertension is manifested in five syndromes [11], of which *Qi*-deficiency is only a minor one. The positive results from this trial were due to the fact that *A. senticosus*, an alternative *Qi*-tonic, was used [16].

Liang *et al*. [54] found that *P. notoginseng *improved endurance time to exhaustion and lowered mean blood pressure in 29 untrained young adults during an endurance exercise. *P. notoginseng *is another important ginseng species classified as homeostatic medicine to arrest bleeding and removes stagnant *Xue*.

## Conclusion

Our analysis of 19 randomized controlled clinical trials of single herb ginseng shows that all the trials with both negative and positive results confirm the specific effects of ginseng indicated by Chinese medicine theory. Therefore, study designs guided by Chinese medicine theory are necessary to validate and improve future randomized controlled clinical trials in Chinese medicine.

## Competing interests

The authors declare that they have no competing interests.

## Authors' contributions

JY conceived the idea of the manuscript. VFE modified the idea and edited the manuscript. YXH and YJ collected references and participated in the discussions. WKG helped draft the manuscript. JY finalized the manuscript. All authors read and approved the final version of the manuscript.
